# A qualitative exploration of the downstream impact of a sustained disruption to the methadone supply in Mexico: effects on the mental, financial, and social health of patients with opioid use disorder in Tijuana

**DOI:** 10.1016/j.lana.2025.101176

**Published:** 2025-07-11

**Authors:** Annick Bórquez, Jesse Lloyd Goldshear, Sheryl Muñoz-Mina, Arturo Tornez, Alicia Harvey Vera, María Elena Medina Mora, Gudelia Rangel, Steffanie A. Strathdee

**Affiliations:** a Division of Infectious Diseases and Global Public Health, University of California San Diego, La Jolla, CA, USA; b Colegio de la Frontera Norte, Carretera Escenica Tijuana-Ensenada, San Antonio del Mar, Tijuana, Baja California, Mexico; c Instituto Nacional de Psiquiatría Ramón de la Fuente Muñiz and Facultad de Psicología/UNAM, Colonia, Huipulco, Tlalpan, Ciudad de México, Mexico; d Comisión de Salud Fronteriza México-Estados Unidos, P.º del Centenario 10851, Zona Urbana Rio Tijuana, Tijuana, Baja California, Mexico

**Keywords:** Methadone shortage, Structural violence, Medications for opioid use disorder, Treatment interruption, Harm reduction, Qualitative

## Abstract

**Background:**

Medications for opioid use disorder (MOUD) are considered the gold standard long-term treatment for opioid use disorder (OUD) as they have a range of health and psycho-social benefits. Sudden reduction or interruptions in MOUD can have both immediate and long-term consequences for patients. We sought to qualitatively examine the impact of the prolonged closure of Mexico’s main methadone production facility (*Psicofarma*) in 2023 on MOUD patients in Tijuana, Mexico.

**Methods:**

We conducted semi-structured qualitative interviews with 20 MOUD patients in Tijuana, Mexico, from May to August 2023. We transcribed interviews and translated them from Spanish to English for analysis. Our analysis followed a thematic and narrative approach, and code congruency was found by consensus. Analytic and narrative memos provided the foundation for the ultimate findings.

**Findings:**

The closure of *Psicofarma* resulted in a sudden stoppage in MOUD for participants. While some were provided with a benzodiazepine alternative and/or psychotherapy by their clinics, most were completely cut off from medical support. Participants reported returning to the illicit market for solutions to both withdrawal and drug cravings. Increased spending on the illicit market drained participant finances and strained employment and familial relationships.

**Interpretation:**

The sudden interruption of MOUD treatment had varied yet predictable effects on our participants. These results demonstrate the need to ensure adequate methadone supply for people with OUD in Mexico and to offer other alternative therapies such as low-threshold buprenorphine.

**Funding:**

Support for this study was provided by 10.13039/100000026NIDA (R01DA049644-S4, T32DA023356 and DP2DA049295).

**Research in context**

**Evidence before this study**

We searched PubMed, Google Scholar, ResearchGate, and ScienceDirect (up to July 1st 2024) for articles documenting shortages in medications for opioid use disorders (MOUD) and their impact on patients' health and wellbeing using the following search terms: *(“methadone” or “buprenorphine” or “opioid agonist therapy” or “opioid substitution therapy” or “medication∗ for opioid use disorder∗”) and (shortage or interruption or disruption)*. We considered qualitative, quantitative research, and research viewpoints as evidence. Relevant articles fell into four main categories: MOUD shortages caused by 1) the COVID-19 pandemic, 2) natural disasters (i.e., hurricanes), 3) political conflicts, and 4) regulatory changes.

Most articles focused on the impact of COVID-19-related disruptions in the U.S., Canada, and a few other countries, highlighting clinic closures and operational challenges, particularly for methadone patients. Mitigation strategies included telehealth prescriptions, increased take-home doses, and regulatory waivers. While some regions adapted quickly, disparities in access (e.g., by race and OUD severity) persisted. Overdose rates in the U.S. rose in the context of an increasingly toxic drug supply and exacerbated social vulnerabilities, making it difficult to estimate the contribution of MOUD disruptions. Studies on MOUD shortages in conflict situations examined the Russian invasion of Crimea (2014) and the war in Ukraine (2022), which severely impacted MOUD access. In occupied Crimea, MOUD was immediately halted, and approximately 20% of patients re-enrolled according to one study. In Ukraine, the two methadone factories shut down, forcing dose reductions and overloaded clinics. Patients often turned to alcohol, pregabalin, or illicit drugs for withdrawal relief. Ukraine’s MOUD system was already under-resourced before the war, and adaptations to address longstanding challenges helped maintain some level of access. Regarding natural disasters, studies focused on hurricanes Sandy, Katrina, and Rita in the U.S. and Puerto Rico, describing emergency MOUD plans such as advance take-home doses, guest-dosing at alternative clinics, and transportation support. Some patients maintained access (especially those on buprenorphine), but others experienced withdrawal and returned to heroin use. Patients with psychiatric conditions or unstable treatment histories were at higher risk of resuming heroin use. Several studies emphasized the need for emergency mental health services for MOUD patients in disaster contexts. Studies addressing MOUD policy shifts included experiences in Canada and the U.S. In British Columbia, methadone formulation changes and the removal of pharmacy delivery services resulted in withdrawal, increased injecting drug use, and reduced HIV treatment adherence. In Ontario, slow-release oral morphine shortages caused higher treatment discontinuation rates. In Maryland, abrupt clinic closures due to legal actions against prescribers led to increased overdoses despite emergency response efforts.

Literature examining the impact of external disruptions to MOUD supply on the lives of patients has mostly documented shortages in the context of unexpected and overwhelming events, including pandemics, hurricanes and wars. These caused severe, but relatively brief or partial disruptions in access, with the exception of the MOUD ban during the Russian invasion of Crimea. Studies investigating treatment interruptions due to regulatory changes, which are most relevant to our study, occurred in the context of alternative medications or treatment settings.

**Added value of this study**

Our study adds to this pool of literature by exploring the downstream impact of a *planned, complete and sustained* disruption to the methadone supply across Mexico, where no alternative evidence-based MOUD was made available and no neighboring treatment settings could welcome patients. While mitigation plans would be expected under such scenario, we describe how this largely preventable disruption caused serious mental, physical, and social harm to MOUD patients, akin to those documented in the context of natural disasters or conflicts, and severely affecting those most marginalised. Arguably, communication and emergency planning were superior in the context of the pandemic and hurricanes described in other studies, as standard operating procedures (SOPs) were put in place.

**Implications of all the available evidence**

Available evidence indicates that disruptions in the supply of MOUD—whether by natural phenomena, conflict or government edict—are harmful to the physical, psychological, and social wellbeing of patients in MOUD treatment. Especially in areas heavily affected by the ongoing overdose crisis, attempts should be made to expand both the availability of these medications and the redundancy of their production or procurement to better protect this population from harm. In addition, emergency plans including SOPs to communicate with patients in advance and support them with take-home doses, transport with alternative treatment sites, and clinical follow-up, should be developed in collaboration with patients and providers based on their experiences. Methadone and buprenorphine are considered Essential Medicines by the WHO, and withholding one medication without offering alternatives is a human rights violation. In Mexico, buprenorphine should be made available to prevent treatment interruptions and expand access.

## Introduction

Medications for opioid use disorder (MOUD), including methadone and buprenorphine, are widely considered to be the most medically and cost-effective long-term treatment for opioid use disorder (OUD)^1^ and are included in the World Health Organisation (WHO)’s list of Essential Medicines. MOUD reduce the risk of fatal overdose, mortality from other causes and HIV and HCV acquisition,^2^, ^3^, ^4^ and improve overall wellbeing.

In Mexico, the availability and dispensing of MOUD is highly limited. This is despite the large number of people with OUD, especially on the northern border, leading to calls by advocacy groups, medical professionals, and policymakers to improve availability and access.^5^ In February 2023, following concerns about quality control in the production of methadone, the Mexican government closed *Psicofarma*, a psychiatric medications’ plant that was the main methadone manufacturing plant in the country.^6^ During the closure, there were no plans in place to provide MOUD clinics with alternative options for treatment, as buprenorphine is unavailable for treating OUD in Mexico by law (see Supplemental File 1 for further information on MOUD policy in Mexico). The order to stop production was sustained until August of 2023, but methadone distribution did not resume immediately, thereby interrupting treatment for hundreds of patients for over seven months in some cases. In 2021, there were 11 public clinics across the country offering MOUD treatment to approximately 400 patients,^7^ and private clinics cover a larger patient population, but a national estimate is unavailable.

In Tijuana—the setting for this study and a city on the Mexico–United States (U.S.) border—approximately 500–1000 patients received methadone in 2022 at the city’s four clinics (one public and three privately operated) based on previous research and discussions with key informants. As one of the busiest land border crossings in the world, Tijuana is situated along a major drug trafficking route and has historically been a hub of both domestic and cross-border drug use. The city also serves as a primary point of return for Mexican citizens deported from the U.S.,^8^ many of whom are left homeless and without access to basic social services.^9^ As one of the cities with the highest rate of illicit opioid use in Mexico, Tijuana is in a unique position to benefit from broad MOUD coverage.

Increased access to low-barrier MOUD is a critical component of addressing substance use health risks and overdose, especially in settings where fentanyl now dominates the opioid supply, such as Tijuana. Beyond the direct medical benefits of MOUD, psychosocial benefits have also been identified.^10^, ^11^, ^12^ In literature dating back to the 1990s, retention in MOUD has been associated with decreased law enforcement contact^13^^,^^14^ and reduced homelessness.^15^^,^^16^ Just as the availability and utilisation of MOUD have demonstrated benefits for patients with OUD, the interruption of these services could have both immediate and long-term consequences.^17^

Given the deliberate nature of *Psicofarma*’s closure and its potential for wide-ranging impacts among MOUD patients in Mexico, we undertook a qualitative study, following a narrative and thematic approach to interviewing and analysis, to document the experiences of MOUD patients in Tijuana both before and during the closure. Indeed, rich, palpable qualitative experiential data can provide a deeper understanding of the links between large socio-structural forces and individual behaviour and experience. Given the sudden closure of *Psicofarma* and its potential impact on the health of Mexico’s MOUD patients, data were collected as rapidly as possible by leveraging and reopening an ongoing cohort study and staff familiar with the social and structural environments of people who use drugs (PWUD) in Tijuana. We aimed to explore 1) experiences of patients receiving methadone in Tijuana after the closure of *Psicofarma* and 2) downstream impacts of this closure on patients’ lives.

## Methods

### Study participants

We recruited 20 participants in Tijuana between 05/23 and 08/23 to take part in a semi-structured qualitative interview examining their experiences with methadone treatment and the sudden closure of *Psicofarma*. Participants were selected among 200 participants recruited leveraging and re-opening an ongoing observational cohort, La Frontera, which studies the cross-border mobility of PWUD, drug supply trends in Tijuana and San Diego, and associated incidence of HIV, HCV, and overdose.^18^ Eligible participants were: 18 years of age or older, living in Tijuana, and had received methadone from a clinic in the city at least once within the past 6 months up to March 2023. Participants were not required to have been active clinic patients at the time of the plant closure, though all were. To recruit these new participants, our study staff spoke with clinic directors, posted study recruitment materials both inside and outside of the clinics, conducted street outreach, and made social media posts. Interested participants were screened for eligibility, consented both orally and on paper, and enrolled the same day, before being interviewed at one of our pre-established field sites. All completed a structured quantitative survey, and a subset of 20 took part in the qualitative interview; five were existing La Frontera participants and 15 were new participants.

#### Study staff information

Interviewers SM and AT are both trained bilingual (Spanish, English) quantitative and qualitative interviewers with expertise in substance use, working as part of La Frontera team since 2020 and living in Tijuana. SM is a physician, and AT has been coordinating and working on public health-related projects since 2008. Co-first authors and coders AB and JLG are both trained research scientists with backgrounds in substance use, social determinants of health, and epidemiology research. AB is a mid-career investigator and JLG is an early-career investigator. Both have training in quantitative and qualitative work. AB is a multilingual native Spanish speaker from Mexico, while JLG is a native English speaker. AB has worked with people who use and inject drugs in Tijuana for the past decade. JLG has lived experience with substance use and addiction.

### Data collection

We conducted 30-min to 1-h, semi-structured interviews in Spanish. Staff interviewed participants in a private area and recorded them on digital audio recorders with informed consent. We uploaded audio files to a secure computer drive on-site and then erased them from the recorders to preserve participant privacy and confidentiality. Interviews followed a common guide structured into broad themes of *drug use history*, *MOUD history and narrative*, *clinic closure experiences*, *clinic closure outcomes*, and *policy recommendations*. Within each domain, interviewers used a variety of probing questions to prompt deeper discussion of participants’ thoughts and feelings. The interview guide can be found in Supplemental File 2. While interviewers were encouraged to follow participant answers with follow-up questions and probes out of order if relevant, most interviews proceeded in a narrative format. Interviews usually began (after the collection of basic demographic information) with questions about the participants’ early lives, introduction to substance use and use narratives, and followed with narrative questions surrounding introduction to MOUD, the course of their treatment, and impact of the closure. The final sample size (N = 20) was determined as thematic saturation was reached and time since the start of the shutdown increased, as we aimed to capture early experiences of treatment interruption. Once we finished conducting all interviews, we transcribed them verbatim in Spanish, deidentified the transcripts, and translated them into English for ease of analysis and presentation. Translation was conducted by AHV, a bilingual native Spanish speaker with expertise in substance use disorders, and familiar with this environment in Tijuana. This preserved the fidelity and integrity of interviewees’ responses. AB re-read the transcripts in Spanish to check the accuracy of translation. Questionnaire responses from the linked cohort study were used to extract demographic data.

### Data analysis

We used Atlas.ti software to conduct this analysis.^19^ We designed a qualitative codebook *a priori* based on the body of literature describing the material, health, and psychosocial benefits of MOUD to patients and included codes specific to the closure and resulting methadone shortage. JLG and AB then conducted an initial reading of the interviews and supplemented the codebook with new additions where relevant. Domains added during the coding process included “Methadone Service Features” and “Circumstances for Methadone Treatment.” The final version of the codebook can be found in Table 1.

**Table 1:** Qualitative codebook for in-depth interviews of patients’ experiences following the methadone factory closure.

Code group	Codes and Subcodes
Substance use	Alcohol
	Benzodiazepines
	Cocaine
	Currently using
	Fentanyl
	Heroin
	Methamphetamine
	Opioid pills
	Other drugs
	Current use
	Past use
Circumstances of methadone treatment	Motivations for using methadone
	Methadone timing
	Methadone timing: First time (Introduction to methadone treatment)
	Methadone timing: Subsequent times
	Methadone timing: Most recent time
Methadone service features	Accessibility
	Accessibility: Distance
	Accessibility: Opening hours
	Accessibility: Cost
	Accessibility: Waiting times
	Staff difficulties
	Staff difficulties: Stigma/Disrespectful
	Staff difficulties: Trying to make money
	Staff Qualities
	Staff qualities: Friendly, respectful
	Staff qualities: Knowledgeable
Health experiences with methadone	Dosing
	Dosing: Adequate dosing
	Dosing: Dosing process
	Dosing: Dosing schedule
	Dosing: Inadequate dosing
	Physical effects
	Physical effects: Craving
	Physical effects: Energy levels
	Physical effects: Overdose experiences
	Physical effects: Side effects
	Physical effects: Withdrawal
	Mental health effects
	Mental health effects: Anxiety
	Mental health effects: Depression
	Mental health effects: Sleep
	Treatment patterns
	Treatment patterns: Duration of episodes
	Treatment patterns: Frequency
	Treatment patterns: Motivations/circumstances for treatment interruptions
	Concurrent use of other substances
	Concurrent use of other substances: Motivations for concurrent use
	Concurrent use of other substances: Other concurrent use effects
	Concurrent use of other substances: Overdose while using concurrently
Attitudes towards methadone	Attitude orientation
	Attitude orientation: Negative
	Attitude orientation: Neutral
	Attitude orientation: Positive
	Attitude perspective
	Attitude perspective: Among Family/Friends
	Attitude perspective: Self
Additional impacts of methadone treatment	Disadvantages of treatment
	Disadvantages of treatment: Boredom
	Disadvantages of treatment: Disconnection from social network
	Disadvantages of treatment: Social stigma
	Disadvantages of treatment: Stigma from doctors
	Methadone was helpful
	Methadone was helpful: Positive impact on social relationships
	Methadone was helpful: Allowed a more structured/functional lifestyle
	Methadone was Helpful: Stabilized finances
Other rehabilitation experiences	Abstinence-based inpatient programs
	Narcotics Anonymous/12 step
	Psychological therapy
	Religious
	Other
Methadone shortage/factory closure	Impressions/feelings about
	Treatment interruption process
	Treatment interruption process: Gradual reduction in dosing
	Treatment interruption process: Sudden reduction in dosing
	Information/support from clinic staff
	Information/support from clinic staff: Advice received about ending treatment
	Information/support from clinic staff: Didn’t know about plant closure
	Information/support from clinic staff: Knew about plant closure
	Information/support from clinic staff: Provision of alternative treatments for withdrawal
	Life impact
	Life impact: Job/Money making
	Life impact: Mental health
	Life impact: Overdose
	Life impact: Physical health
	Life impact: Relationships
	Solutions (self-identified)
	Solutions (self-identified): Other drugs
	Solutions (self-identified): Other medications
	Solutions (self-identified): Other rehabilitation options
	Advice to government

JLG and AB followed a combined thematic and narrative approach to interview analysis.^20^^,^^21^ This combined approach was achieved in two steps. First, we each started by thematically coding a sub-sample of three interviews, compared our findings, and modified the codebook a final time. We then used the finalised codebook to analyse the remaining 17 interviews each, also thematically, adding memos and analytic notes as necessary. Once all interviews were coded, we merged our separate analysis files and began reviewing our findings. In the second step, we used the narrative structure of the interviews themselves and our thematic codes to track participant experiences across early MOUD treatment, recent MOUD treatment pre-closure, and experiences post-closure. While we did not construct individual participant timelines, this combined approach was useful in delineating common sequences of events among the sample. Where our code application or analysis diverged, we discussed until reaching consensus. This consensus coding approach improves the validity of our results, but yields no useable metric of interrater reliability.^22^ After recording final combined analytic notes, we chose representative quotes and used appropriate pseudonyms to protect participants’ confidentiality.

Here, we have chosen to present our findings in a broadly narrative order with representative quotes from participants. In addition to participant pseudonyms, quotes are also accompanied by participant demographic information for added context. Additional quotes not included in the Results are presented in Supplemental File 3. In reporting these results, we have adhered as closely as possible to COREQ guidelines (Supplemental File 4).^23^

All study procedures were approved by the Institutional Review Boards at both UCSD and University of Xochicalco.

### Role of the funding source

Study funders had no role in study design, data collection, data analysis, interpretation, writing of the report or decision to submit.

## Results

### Participants’ background

Of the 20 participants interviewed, 9 identified as women and 11 as men. All participants were cisgender based on reports of gender identity and sex assigned at birth in the quantitative survey. The mean age was 41.8 [SD = 11.6]. The majority were from Tijuana, all identified as Hispanic, Latino, Mexican or Chicano and 55% had been deported from the US (refer to Table 2). They had been using heroin for 22.5 years on average [SD = 12.4]. Participants had been on methadone treatment between one and 6 times, for a duration of 2 months up to 5 years. They had their first experience of methadone treatment on average 13 years ago (SD = 10.7), in some cases in the U.S., while others had started for the first time in 2023 prior to the plant closure. At the time of the interview, 5–8 months had passed since participants had last received methadone.

**Table 2:** Demographic and opioid use disorder (OUD) treatment information at time of interview.

Sociodemographic characteristics		
	Mean (SD) or N (%)	Range
Age (Years)	41.8 (11.6)	22–57
Gender		
Man	11 (55%)	
Woman	9 (45%)	
Hispanic/Latino/Mexican/Chicano ethnicity	20 (100%)	
Ever deported from U.S.	11 (55%)	

**Methadone** treatment characteristics		
	Mean (SD) or N (%)	Range
First methadone treatment in U.S.	1 (5%)	
Years since first heroin injection (Years)	22.5 (12.4)	3–39
Years since first methadone treatment enrollment	12.9 (10.7)	1–31
Times enrolled in methadone treatment	2.8 (1.5)	1–6
Methadone cost per daily dose (USD)	8.80 (2.6)	2.0–11.8
Methadone clinic at last treatment		
Public	8 (40%)	
Private	12 (60%)	
Months since start of closure at time of interview	6 (1)	5–8

Several participants shared histories of childhood trauma and family substance use, while others were introduced to opioids through peers or by romantic partners. All participants had a history of polysubstance use, generally involving opioid and stimulant use (mostly methamphetamine).

### Introduction to methadone

Participants commonly reported hearing about methadone for the first time while detained at Tijuana’s municipal detention center. Others reported hearing about it through peers who were on treatment themselves or through family members. Some mentioned dismissing the information at first but reconsidering after seeing peers doing well or after failing to stop using heroin through abstinence-based programs or other methods. Eventually, participants all started MOUD treatment at one of the available clinics, where they were usually informed about its legal status, effectiveness compared to their typical street opioid use, and introduced to the dosing protocol and schedule.

*“At the clinic they explained to us, the first time they brought the juice [methadone], Dr Romero [pseudonym] was the first one to bring it to Tijuana and [said] that it was used during the 2nd World War and it was given to soldiers so they would feel strength and courage, to feel good … that is a sensation similar to heroin … and that it is legal and only one dose a day is better”* – Santiago (Male, 55)

### Motivations to use methadone

When asked about the context in which they decided to initiate methadone treatment and their motivations to do so, some spoke about their situation being unsustainable given poor health and living conditions. Others said they had urgent financial needs as they had a family to support and most of their earnings were spent on heroin. Several mentioned the fear of losing their family. Others felt at high risk of resuming heroin use, and one participant said she sought treatment after her partner was incarcerated, as he represented the main reason and gateway for her to use heroin.

*“Because I was working, I had a pregnant wife, uh, expenses at home. And I was not making ends meet … I had to, well, honestly, steal and such, but I got to know methadone and well, yes, it does help, because uh, with heroin, one starts using it for fun, but then it’s no longer fun because you have to worry about how to pay for the heroin which … uh it physically wears you out”. –* Roberto (Male, 41)

### Experiences of methadone pre-closure

Participants expressed positive attitudes towards methadone, in terms of integrating the medication dispensing process into their routine and its effect on their physical and mental health and on other important aspects of their lives. Even given the limited availability of methadone in Tijuana pre-closure, frequent changes in clinics’ locations and occasional transit barriers identified in interviews, participants viewed clinics and their staff positively. Many felt that the staff were kind, conscientious, and that the clinicians in charge provided adequate dosing regimens.

*“Well, there was a girl […], and she’s a very kind person to me, someone who understood you, supported you, someone who asked you every day how you were feeling apart from the doctor. […] I could see that they were interested in us feeling well, that the methadone was truly helping us.”* – Andrés (Male, 57)

When asking participants if they were using other licit or illicit drugs in conjunction with methadone, the majority said they only used methadone, while a few reported smoking marijuana and drinking alcohol, and two reported using heroin alongside for a period.

*“In the first few days, yes, I was still using drugs, but as the days went by, I started reducing the consumption until I got used to it here, and then I only took methadone.”* – Antonio (Male, 49)

Regular MOUD treatment enabled participants to gain steady employment, improve their finances, repair and maintain important social relationships and regain their sense of self. One participant felt that:

*“Methadone users get used to living this way. We use it to live a good life. Heroin won’t allow you to do what methadone does. For me, it has worked. With methadone, I can pay my rent, electricity, water bills, and I can have a family.”* – Román (Male, 55)

### Post-closure

Given the positive impact of MOUD on interviewees’ lives, news of the sudden closure was frustrating to all our participants. They unanimously indicated that no advance notice was given. In the best-case scenarios, participants’ clinics remained open and were able to provide a benzodiazepine, usually *clonazepam*, that could mitigate withdrawal symptoms and potentially help reduce subsequent cravings. Some participants reported receiving placebos and/or psychotherapy.

Most described their clinics as either closing completely or remaining open but unable to provide a methadone alternative. In several instances, interviewees arrived in the morning at their usual clinics only to find the doors closed.

*“I was there until this January [2023] when we arrived, like any other day, and found it closed. They didn’t give us any explanations, reasons, or indicate when it might reopen. As of now, it remains closed.* […]*Yes, I’ve searched online, in pharmacies, clinics, but it seems from what everyone tells me, it’s not available in Mexico.”* – Rodrigo (Male, 25)

In all cases, the supply of methadone was constrained by the closure, and participants immediately experienced withdrawal symptoms.

The closure of *Psicofarma* and the subsequent constraint on the methadone supply in Tijuana had a range of health effects on participants. Most immediate was the anxiety experienced by participants upon learning that they had been cut off from MOUD and would have to suffer the effects of withdrawal. For some, this anxiety was difficult but manageable. For many, however, the accompanying opioid cravings were all-consuming. In cases where clonazepam was provided as an alternative, participants often described it as offering only temporary relief.

*“They did give us some medication … they don’t do anything, you know? … And when you’re going through withdrawal, you don’t want to know about anything else. You don't want to talk, what I want is to get well (ease withdrawal symptoms), I want the cravings and desperation to go away. I want to get back to normal, you know what I mean? … I have this mental chaos going on.”* – Román (Male, 55)

Some participants described eventually returning to the illicit drug market for a range of self-identified solutions, including increased cannabis consumption, benzodiazepines, and stimulants like cocaine. Primary reasons for use included reducing anxiety, warding off withdrawal symptoms, and distracting from opioid cravings.

*“Yes, I hope not to fall back [resume heroin use], but as long as methadone is available here, because if there’s no methadone, there’s nothing else that can replace the heroin anxiety like methadone … And right now, since there’s no methadone, I say, well, I have to use [benzodiazepine] pills, two pills.”* – Teresa (Female, 48)

Others, however, returned to heroin and/or fentanyl use after being cut off from MOUD. None reported overdose experiences following the clinics’ closure. However, they did mention changes in the supply, unavailability of heroin and fear of fentanyl.

*“On May 21st, 22nd, and 23rd, that’s when I started feeling the withdrawal, and I immediately went to the “puerta negra” (the black market), but it wasn’t methadone. I had to go to the canal [open drug market] to find something, and since May 20th, 2023, I’ve been using heroin again. It’s been almost two and a half months now.*” – Román (Male, 55)

Return to the illicit drug market post-closure resulted in several related material and social outcomes for participants. Notably, methadone in Tijuana had been relatively inexpensive, with participants paying anywhere from $50–$150 MXN (i.e., 2.7–8 USD) per daily dose and some being offered reduced prices based on a financial assessment. The illicit market was vastly more expensive, and many of our participants described this increased spending as a drain on their financial resources and ultimate stability.

*“When I do [cocaine], especially when I’m feeling really bad, I’ve probably spent around MXN $1500* (i.e., 80 USD)*, as a conservative estimate. It’s affected me at work because sometimes I don’t feel in the right state to work and I miss days. I don’t think it’s helping me, using these substances to feel more relaxed.”* – Omar (Male, 29)

Finding and purchasing drugs on the illicit market also took a great deal more time than the routine trip to the methadone clinic. Participants noted that this increased time and money spent procuring drugs was time not spent at work (as noted by Omar above) or maintaining familial relationships—many of which were already tenuous and strained by prior drug use. Several described their families as being supportive of their continued MOUD treatment, but as they returned to the illicit market, this support was often withdrawn. One participant described the loss of trust from their family members as stressful.

*“When I started with marijuana, that’s when my panic set in … my family is becoming overprotective again, always watching over me to see what I’m doing, where I’m going, knowing that I’m not taking the medication … maybe that’s why … It stresses me out knowing my family doesn’t trust me, to the point that I don’t trust myself either …”* – Leticia (Female, 22)

The combination of increased spending on drugs and lack of time spent at jobs and with family resulted in a loss of employment, housing, or both for some participants. This return to the illicit drug market was also often accompanied by fear and recognition of falling back into ‘the life’ of street drug purchasing and use.

*“Right now, I’m really struggling because there’s no methadone. I’ve already lost my partner due to this situation. And I’m on the verge of losing my job; they told me they’ll do a drug test, and I don’t know what I’ll do. But I hope the clinic opens again before things get worse. I wish someone would do something for the clinics, really, because I’m not the only one going through all this. Many people have lost everything we achieved—the car, the partner—it’s sad to go back to heroin, to go back to the streets, to end up in the canal again. We’ll all be [living homeless] in the canal again.”* – Román (Male, 55)

A few participants displayed resilience in the face of these changes. Support of family in maintaining housing and income and higher social status seemingly made it easier to overcome the challenges inherent to the plant closure. In one case, a participant had been paying for psychological therapy.

*“Yes, I’ve tried seeking support from a psychologist, but no matter how hard you try, it’s very challenging. Methadone helped me a lot, a lot … Imagine now, I’m spending on another addiction, which is marijuana, and I’m also paying for therapy just to somewhat keep going because it’s so addictive.”* – Rodrigo (Male, 25)

### Change in opinion about methadone among participants following the methadone shutdown

We investigated the possibility that methadone dispensing interruptions could change participants’ sense of trust and confidence in MOUD, which could affect their willingness to re-initiate treatment in the future. However, participants unanimously said their views on methadone had not changed and that they would re-initiate treatment if it became available.

*“No, no, no, no, it hasn’t changed my mind, but … it was more of a shock … I was scared when I saw that place closed … I thought I want to stay like this, but no, I have no doubts”* – Daniel (Male, 29)

### Policy recommendations from participants

Finally, we asked participants to share policy advice or recommendations in relation to the methadone shutdown to get their views on this issue beyond their individual experiences. Most participants chose to reiterate the personal impact of MOUD treatment on their lives and the potential for it to positively influence the lives of others.

*“I would tell the Mexican government that it would be a good option to bring back methadone because there are many people, like me, who have this problem of addiction, and that is a great help, to put up those clinics, yes, it helps a lot because you arrive at the clinic, you do what you have to do, pay, and they give you your medication and you go back to work, you are a normal person. You go back to being like any other person.”* – Eduardo (Male, 57)

Beyond the personal impact of the closure, some participants chose to emphasise the preventive value of MOUD availability, framing its wider use to reduce both crime and exposure to emerging, novel street drugs. Participants attributed the “delinquent” behaviour they engaged in to their OUD and vice-versa, and framed a recommendation for increased MOUD availability as a healthy way to reduce both.

*“Well, I would say that they should obtain methadone again and distribute it, because people will always resort to new drugs, and new drugs are constantly emerging. It would be a way to reduce delinquency, as it was the same thing that led me to use heroin again. So, in that way, it would help reduce drug consumption. Yes, it is necessary to bring back that treatment as a support to stay away from drugs.” –* Karina (Female, 36)

## Discussion

We reported on the experiences of patients receiving MOUD in Tijuana, Mexico, following the methadone shortage that affected the entire country between February and August 2023 due to the sudden and prolonged closure of *Psicofarma*. Our findings indicate it had considerable physical, psychological, material and social impacts on patients, who were essentially cut off from their methadone treatment regimens until late in the year, even after production had resumed. Many interviewees described using other drugs like cannabis and benzodiazepines to cope, while others eventually resumed opioid use. The increased price of other drugs compared to methadone drained their finances and strained familial relationships. In the worst cases, participants described losing employment and housing stability. We observed that (limited) resilience to these effects relied on strong family support in both housing and monetary.

While momentous, this is only one of five interruptions in methadone availability over the past decade in Mexico, all of which were related to logistical or regulatory processes involving methadone importation or production.^24^ In this respect, these shortages were fully preventable events that compromised patients’ lives, but were, to a large extent, invisible to the media and did not cause national or international outrage. This event is best understood through the lens of *structural violence*, which we define (based on the definitions of both Galtung and Brandy X. Lee), as *an avoidable impairment of human needs placed on groups of people by institutions of power that forcibly limits or constrains their quality of life*.^25^^,^^26^ In agreement with other scholars, we believe that structural violence should be addressed as a public health issue that serves as an overarching cause of premature death and unnecessary disability.^27^ Structural violence as described by Galtung is “as natural as the air around us”, in the sense that it is so ingrained in our social systems that it becomes normalised.^28^ The stigma associated with substance use disenfranchises this patient population, both at the individual and social level, leading to public indifference in the face of an irrefutable violation of human rights. For the majority of our participants, methadone treatment represented a conduit back to a “normal” life and fulfilment of a variety of basic human needs. They gained stability while using methadone instead of street opioids and were able to maintain shelter, employment, and positive social ties. The sudden, intentional closure of *Psicofarma* represents a form of structural violence that forcibly affected the lives of MOUD patients in Tijuana. While participants did not protest or express indignation, when asked to share policy recommendations, they had a consistent message to convey: methadone constitutes a concrete solution to regain stability across multiple aspects of life and the government should invest in it to address OUD and its associated health and social impacts.

These findings are consistent with the literature on both the psycho-social benefits of MOUD and the impacts of its interruption on the lives of patients.^29^ Of note, participants described these benefits as occurring even within the already-constrained MOUD environment of Tijuana. Few comparable long-term methadone supply shortages have been documented in the literature, the most recent being changes to the U.S. methadone supply during the early COVID-19 pandemic.^30^ During 2020, the per-capita supply of methadone dropped, and in June 2021 had not recovered to 2019 levels.^30^ One comparable situation occurred when Russia annexed Crimea in 2014. Methadone was immediately banned in accordance with Russia’s federal law, affecting over 800 people receiving treatment.^9^ Although a formal evaluation was not conducted, within a year, an estimated 80–100 people had died, some due to overdose or suicide.^10^ Along with these findings, ours provide valuable information about the immense significance of this medication for people who want to remain in treatment (in contrast to studies comparing experiences of people in and out of treatment out of choice).

The experiences of participants in our study are troubling in the context of high fentanyl penetration in the Mexican drug market. Across Mexico, and specifically in Tijuana, drug overdose incidence has risen,^31^^,^^32^ although surveillance data are limited.^33^ Return to the current illicit market for fentanyl-naïve patients poses serious risks of fatal overdose, especially since naloxone availability is extremely limited in Mexico, given its unwarranted status as a psychoactive substance.^34^

Several limitations warrant consideration. As with all interview-collected qualitative data, our results may be subject to social desirability bias. Interviewees may also have been biased towards positive recall of their MOUD treatment due to cravings experienced at the time of their interviews and because, in the face of the methadone shortage, an imperfect program appears much more desirable than an absent one. Our data may also have been biased towards patients who were better able to cope following the shutdown and therefore easier to reach by our team. In particular, the participants we interviewed had not yet experienced overdoses following treatment interruption, and our study could not have captured those who died from overdose shortly after the closure. While qualitative studies do not seek to achieve population representativeness, we note that we oversampled women to better capture their experiences. Participants were otherwise representative of people with OUD in Tijuana in terms of ethnicity and deportation experiences. Importantly, a more complete account of the closure would require including the views of clinics’ staff and patients’ family members. Regrettably, one of the private methadone clinics did not recover from the shutdown and has closed indefinitely. Our study is also limited to Tijuana, while patients across the country were affected by the closure. Nonetheless, we believe it is an important contribution towards documenting the experiences and consequences of this impactful policy decision.

As one of the few studies of MOUD patients in Latin America, our study brings further attention to the needs of people with OUD across the region. A 2022 joint United Nations Office on Drugs and Crime and WHO report covering six countries (Bolivia, Dominican Republic, Ecuador, Guatemala, Mexico, and Panama) found wide variation in substance use disorder treatment provision.^35^ Of the 254 facilities surveyed in Mexico, 30% indicated the ability to prescribe and/or dispense methadone. This was high compared to Dominican Republic (5% prescribing and 2% dispensing) and Guatemala (17%), and low compared to Ecuador (69% and 67%, respectively). In a separate study, authors in Colombia reported that while methadone was available in areas with the highest prevalence of opioid use, many barriers to effective treatment were still present.^36^ They recommended expanding the national methadone supply and importing other medications such as buprenorphine and naltrexone. While overall opioid use across the region is low, demand for treatment among people with OUD may be high.

These findings should also be contextualised within the broader impact of both Mexico’s and the nearby U.S.’ violent “war on drugs” and the added violence of the production and trafficking of drugs. While Mexican drug policy is—at least formally—aligned with a public-health-oriented approach where drug possession in small quantities is depenalised and evidence-based treatment recommended since 2009, implementation has been inconsistent at best, and the cycle of mass violence against PWUD continues.^33^ These dual sources of violence have led to ever-increasing militarisation, forced displacement of communities, a steep rise in homicide rates and ‘missing people’, and the uncovering of mass graves. In this environment, both mandated and voluntary treatment for substance use disorders remain mostly unregulated and dominated by abstinence-based programs.

Within this context of structural violence, our findings demonstrate the need for both wider availability of MOUD in Mexico and stability in its supply. While plans were in place to support an expansion of MOUD provision, the federal government of Mexico has, to this point, not implemented them.^37^ Mexico should seize this opportunity to not only rectify its methadone shortages, but to improve drug treatment by diversifying its production sources or importing it, and making buprenorphine available, including in its long-acting injectable and depot formulations. Importantly, the proposed General Health Law reform, which would remove naloxone from the list of psychoactive drugs needs enacting to allow for the scale-up of much needed overdose education and naloxone distribution programs. In the very short term, a solid national methadone shortage preparedness plan that clearly lays out protocols to support patients during the transition is urgently needed and should be made available to all clinics. Amidst other forms of structural violence affecting PWUD in Mexico, including acute cartel violence, police militarisation and a migration crisis, reliable access to evidence-based treatment is the bare minimum that should be warranted by the state.^38^, ^39^, ^40^

## Contributors

AB and SAS designed the study and obtained funding support, AB developed the topic guide for the qualitative interviews, AT and SM led the qualitative interviews, AHV coordinated and supervised the field work, AHV translated the interview transcripts, JLG and AB coded and analyzed the interviews, JLG wrote the first draft of the manuscript. AB, JLG, SMM, AT, AHV, MEMM, GR and SAS contributed to writing, reviewing and editing the manuscript.

AB and JLG had full access to the data and verified it. AB, JLG and SAS were responsible for the decision to submit the manuscript.

## Data sharing statement

Due to the sensitivity of the data and participant privacy concerns, the qualitative data are not available for public sharing. However, interested researchers may contact the corresponding author, [AB], for potential access with appropriate ethical approval.

## Declaration of interests

Authors have no conflicts of interest to declare.

## References

[1] Onuoha E.N., Leff J.A., Schackman B.R., McCollister K.E., Polsky D., Murphy S.M. (2021). Economic evaluations of pharmacologic treatment for opioid use disorder: a systematic literature Review. Value Health.

[2] Wakeman S.E., Larochelle M.R., Ameli O. (2020). Comparative effectiveness of different treatment pathways for opioid use disorder. JAMA Netw Open.

[3] Gowing L., Farrell M., Bornemann R., Sullivan L.E., Ali R. (2008). Substitution treatment of injecting opioid users for prevention of HIV infection. Cochrane Database Syst Rev.

[4] Platt L., Minozzi S., Reed J. (2017). Needle syringe programmes and opioid substitution therapy for preventing hepatitis C transmission in people who inject drugs. Cochrane Database Syst Rev.

[5] Graham T. Carriers sneak life-saving drugs over border as Mexico battles opioid deaths. The Guardian. https://www.theguardian.com/global-development/2024/jan/23/mexico-us-overdose-drug-naloxone-fentanyl-opioid.

[6] Strathdee S.A., Goodman-Meza D., Rafful C.M. (2023). Addressing opioid use disorder: Mexico’s step backwards. Lancet Reg Health Am.

[7] Varela J., Ramirez H., Sirot G. (2021).

[8] Unidad de Política Migratoria (2025).

[9] Contreras Velasco O. (2016). Vivir en los márgenes del Estado: Un estudio en la frontera México-Estados Unidos. Reg Sociedad.

[10] Chang H.-M., Huang M.-C., Fang S.-C., Lin S.-K. (2023). Quality of life and associated factors of heroin-dependent patients receiving methadone and buprenorphine maintenance treatment. Neuropsychopharmacol Rep.

[11] De Maeyer J., Vanderplasschen W., Camfield L., Vanheule S., Sabbe B., Broekaert E. (2011). A good quality of life under the influence of methadone: a qualitative study among opiate-dependent individuals. Int J Nurs Stud.

[12] Ponizovsky A.M., Margolis A., Heled L., Rosca P., Radomislensky I., Grinshpoon A. (2010). Improved quality of life, clinical, and psychosocial outcomes among heroin-dependent patients on ambulatory buprenorphine maintenance. Subst Use Misuse.

[13] Bell J., Hall W., Byth K. (1992). Changes in criminal activity after entering methadone maintenance. Br J Addict.

[14] Russolillo A., Moniruzzaman A., McCandless L.C., Patterson M., Somers J.M. (2018). Associations between methadone maintenance treatment and crime: a 17-year longitudinal cohort study of Canadian provincial offenders. Addiction.

[15] Royse D., Leukefeld C., Logan T.K. (2000). Homelessness and gender in out-of-treatment drug users. Am J Drug Alcohol Abuse.

[16] Orwin R.G., Scott C.K., Arieira C. (2005). Transitions through homelessness and factors that predict them: three-year treatment outcomes. J Subst Abuse Treat.

[17] McGlothlin W.H., Anglin M.D. (1981). Shutting off methadone: costs and benefits. Arch Gen Psychiatry.

[18] Strathdee S.A., Abramovitz D., Harvey-Vera A. (2021). Prevalence and correlates of SARS-CoV-2 seropositivity among people who inject drugs in the San Diego-Tijuana border region. PLoS One.

[19] (2019). ATLAS.ti (Version 8.4.15).

[20] Clarke V., Braun V. (2017). Thematic analysis. J Posit Psychol.

[21] Butina M. (2015). A narrative approach to qualitative inquiry. Clin Lab Sci.

[22] Cascio M.A., Lee E., Vaudrin N., Freedman D.A. (2019). A team-based approach to open coding: considerations for creating intercoder consensus. Field Methods.

[23] Tong A., Sainsbury P., Craig J. (2007). Consolidated criteria for reporting qualitative research (COREQ): a 32-item checklist for interviews and focus groups. Int J Qual Health Care.

[24] Romero-Mendoza M., Peláez-Ballestas I., Almanza-Avendaño A.M., Figueroa E. (2022). Structural violence and the need for compassionate use of methadone in Mexico. BMC Public Health.

[25] Lee B.X. (2019). https://www.wiley.com/en-us/Violence%3A+An+Interdisciplinary+Approach+to+Causes%2C+Consequences%2C+and+Cures-p-9781119240686.

[26] Ho K. (2007). Structural violence as a human rights violation. Essex Human Rights Rev.

[27] Farmer P.E., Nizeye B., Stulac S., Keshavjee S. (2006). Structural violence and clinical medicine. PLoS Med.

[28] De Maio F., Ansell D. (2018). “As natural as the air around us”: on the origin and development of the concept of structural violence in health research. Int J Health Serv.

[29] Tenore P.L. (2008). Psychotherapeutic benefits of opioid agonist therapy. J Addict Dis.

[30] Chen A.Y., Powell D., Stein B.D. (2022). Changes in buprenorphine and methadone supplies in the US during the COVID-19 pandemic. JAMA Netw Open.

[31] Rivera Saldana C.D., Abramovitz D., Meacham M.C. (2021). Risk of non-fatal overdose and polysubstance use in a longitudinal study with people who inject drugs in Tijuana, Mexico. Drug Alcohol Rev.

[32] Bailey K., Abramovitz D., Patterson T.L. (2022). Correlates of recent overdose among people who inject drugs in the San Diego/Tijuana border region. Drug Alcohol Depend.

[33] Bejarano Romero R., Arredondo Sánchez-Lira J., Slim Pasaran S. (2023). Implementing a decentralized opioid overdose prevention strategy in Mexico, a pending public policy issue. Lancet Reg Health Am.

[34] Camhaji E. (2023, December 17). https://elpais.com/mexico/2023-12-17/el-veneno-el-antidoto-y-el-infierno-un-grito-de-auxilio-desde-la-zona-cero-de-la-epidemia-de-fentanilo.html.

[35] (2023). Treatment services for substance use disorders in Latin American countries: findings from the UNODC-WHO facility survey for field testing (2203666; p. 82).

[36] González G., Giraldo L.F., DiGirolamo G. (2019). Facing the growing heroin problem in Colombia: the new methadone-assisted treatment programmes. Rev Colomb Psiquiatr.

[37] Moreno J.G.B., Licea J.A.I., Ajenjo C.R. (2010). Tackling HIV and drug addiction in Mexico. Lancet.

[38] Deschak C.I., Infante C., Mundo-Rosas V., Aragón-Gama A.C., Orjuela-Grimm M. (2022). Food insecurity and coping strategies in international migrants in transit through Mexico. J Migr Health.

[39] Morales F.R., Nguyen-Finn K.L., Haidar M., Mercado A. (2022). Humanitarian crisis on the US-Mexico border: mental health needs of refugees and asylum seekers. Curr Opin Psychol.

[40] Lechuga J., Ramos R., Dickson-Gomez J. (2023). Institutional violence from police militarization and drug cartel wars as a ‘Big Event’ and its influence on drug use harms and HIV risk in people who inject drugs on the U.S.–Mexico border. Int J Drug Pol.

