# Supplementary material for: A qualitative exploration of the downstream impact of a sustained disruption to the methadone supply in Mexico: effects on the mental, financial, and social health of patients with opioid use disorder in Tijuana
Source: Lancet Reg Health Am. 2025 Jul 11;49:101176. doi: 10.1016/j.lana.2025.101176 (PMC12274902; doi:10.1016/j.lana.2025.101176)
Supplement: Supplemental Files 1–4 [file mmc1.pdf]

Supplemental File 1.

Medications for Opioid Use Disorders Policy Snapshot in Mexico

The methadone shortage described in our manuscript is the fifth national-level lengthy interruption of methadone availability in the past decade in Mexico. Here, we provide a brief overview of the circumstances behind these shortages, including the Mexican prescription opioid regulatory framework, the undersupply of prescription opioids in Mexico and the broader persistent medication shortage issues experienced at national level (including for cancer, HIV and other serious conditions).

While buprenorphine is available as an analgesic/pain relief medication in Mexico, it has not been approved as a treatment option for OUD[1]. Medications in Mexico are regulated by COFEPRIS (i.e. the "Federal Commission for the Protection against Health Risks") and buprenorphine is classified as a "Class II" medication under article 226 of the "General Health Law"[2], meaning that it is a psychotropic that can only be prescribed by surgeons, veterinarians and dentists in the context of analgesia/pain relief using a special license[3]. Methadone is classified as a "Class I" medication (alongside fentanyl) and is therefore prescribed under even stricter conditions, but its use to treat OUD was approved by COFEPRIS in 1992.

While there are some advocacy efforts in Mexico to remove regulatory barriers to buprenorphine for OUD, these are diluted among strong cautionary tales and regulatory efforts to prevent a prescription opioid epidemic, as experienced in the U.S. and Canada. These efforts include calls to classify buprenorphine as a "Class I" medication to further monitor its prescription[4].

In the context of strict regulation of opioid prescription for pain and of opioid medication shortages, Mexico faces an under-prescription of opioids to treat pain. Bills to increase the availability of opioids for pain relief have been proposed, including some to regulate poppy plantations in Mexico to have control over the production of medications, prevent shortages and satisfy the demand[5]. There are several factors in play, and OUD is not considered a priority given low (and likely under-) reported rates at national level (albeit high in specific settings), and therefore, policy change is slow and inefficient (e.g. naloxone still considered a psychotropic drug despite active legal initiatives to make it available over the counter). Given the very tight regulations around opioids' prescription (leading to apparent low demand) and production, few national pharmaceutical companies are willing to invest to produce them, effectively creating a monopole and exposing the system to shortages[6]. While the health system could rely more on exports to prevent shortages, cost likely represents a barrier, but lack of political will in particular, has led to stagnation on this front. Importantly, shortages of methadone and other prescription opioids are happening in the context of acute large-scale medication shortages in Mexico, which have been documented more systematically since 2022 by "Colectivo Cero Desabasto", an initiative aiming to generate evidence and accountability about this issue to identify solutions.[7] While difficult to characterize due to the health system's fragmentation into multiple institutions, based on the number of reports submitted to the initiative, medication shortages in 2023 were found to have primarily affected patients needing medications for mental health issues.[7] Vaccine coverage (e.g. measles/mumps/rubella, tuberculosis) has also been found to decline. Medication shortages are attributed to poor decision-making around their procurement and distribution at high administrative levels.

References

1. Villafuerte García Adriana, Hernández Martínez Luis Javier, Domínguez Ruíz Ángel, Guzmán Karla Mayte, Rafful Claudia. Opioides, sobredosis y los desafíos de la reducción de daños como estrategia eficiente en el campo de la salud pública. Rev. Fac. Med. (Méx.). 2024 Ago [citado 2025 Jun 06] ; 67(4): 43-56. (http://www.scielo.org.mx/scielo.php?script=sci_arttext&pid=S0026-17422024000400043&lng=es).
2. COFEPRIS, 2024. Listado Actualizado de Medicamentos de Referencia 2024/02 (https://www.gob.mx/cms/uploads/attachment/file/945072/LMR_2024-02_actualizaci_n_06_septiembre_2024.pdf)
3. Rivera A. 2023. *¿Qué se necesita para prescribir medicamentos controlados? - Medscape - 29 de agosto de 2023 (*https://espanol.medscape.com/verarticulo/5911350#vp_1)
4. Covarrubias-Gómez Alfredo, Templos-Estebán Luz A, Esquer-Guzmán Héctor M, Carrillo-Torres Orlando, Pavón-Sánchez Rodrigo A, Arriaga-Morales Ernesto et al . Reflexiones sobre el uso de opioides para el tratamiento del dolor crónico no-oncológico en México. Rev. mex. anestesiol. [revista en la Internet]. 2019 Dic [citado 2025 Jun 06] ; 42(4): 312-314. (http://www.scielo.org.mx/scielo.php?script=sci_arttext&pid=S0484-79032019000400312&lng=es).
5. Iniciativa que reforma los artículos 194 y 198 de la let general de salus y 198 del código penal federal, a cargo del diputado Manuel Huerta Martínez, del grupo parlemntario de Morena.https://sil.gobernacion.gob.mx/Archivos/Documentos/2019/02/asun_3809312_20190207_1543326147.pdf
6. Hernández Ojesto LA. and Linares A., 2023. Intentan vencer la adicción a los opioides en México y no encuentran la medicina que necesitan (https://www.telemundo.com/noticias/noticias-telemundo/salud/hablan-los-afectados-por-la-escasez-de-metadona-en-mexico-no-se-que-ha-rcna87955)
7. *Toche, Nelly, 2024. Radiografía del Desabasto de medicamentos en México: "7,5 millones de recetas no surtidas en 2023" - Medscape - 10 de jun de 2024.(Radiografía del Desabasto de medicamentos en México: "7,5 millones de recetas no surtidas en 2023")*

Supplemental File 2

<u>Topic guide</u>

<u>Qualitative Interview Guide to Explore Methadone Treatment Interruptions in Tijuana.</u>

First experience with methadone

Please tell me about your first experience with methadone treatment in Mexico. How did you find out it existed and why did you decide to take it? Did you have any beliefs or fears about methadone before using it for the first time? Where and when did you first receive it, and how did it work for you? (Probe: How long did you receive methadone for?)

What prompted you to stop methadone that time? How did it go that time? [Inquire: Did you suffer from withdrawal or use heroin again?]

Next experiences with methadone

After that first time, do you remember how many more times you went back into methadone treatment?

Last experience with methadone

Tell me about the last time you resumed methadone treatment.

Motivations/Circumstances to restart: Inquire: When was it? Why did you decide to try again?

Access to treatment: Did you have any difficulty getting treatment? How did you organize yourself to go regularly to receive your treatment? [schedules, transportation, how they coordinated it with other responsibilities]. Could you describe what the process was to receive your dose? [inquire: how long it took, whether they received psychological therapy or they simply gave them the treatment]

Pharmacological impact: How did you feel with the dose you received? [Was it too low, too high, appropriate?] Was it difficult to identify the dose that worked for you? What other medications or drugs did you use while receiving methadone? How did your use of these other drugs change compared to before starting treatment? Did you suffer from side effects?

Did you ever overdose during that period? Tell me about those experiences, especially the last time it happened to you. (Probe: Were you receiving methadone at that time or had you stopped using methadone? If you had stopped using methadone at that time, was it your decision to stop?)

Socio-economic and relational impact: What was a typical, common day like when you were on methadone? Did you feel that methadone was helping you? How? Did using methadone (compared to heroin) have any impact on your finances?

How did it impact your work or your relationships with your family, partner or friends? Who knew you were taking the treatment? What did they think? For you, what were the main advantages and disadvantages of methadone?

Tell me about your experiences with other drug rehab programs or support groups (such as Christian or NA groups) that you have attended while receiving methadone. How did they complement the treatment, if at all?

Treatment Discontinuation: Tell me about the last time you stopped using methadone for more than a day. (Probe: Did you decide to stop using methadone or was something preventing you from accessing methadone?) Tell me when this happened and what your life has been like since then. (Probe: How long have you stopped using methadone since the last time?) How has this decision affected you? (Probe: in terms of your physical health, your spirits, your work, your finances)

If the person mentions the shortage or the fact that the treatment center told them that methadone was no longer available: How has the methadone shortage affected your ability to get methadone? What is your current dosing schedule? How did the methadone clinic deal with the shortage? (Probe: Did they give you any medication to deal with withdrawal and other effects associated with stopping treatment (such as insomnia?)).

Whether or not participant mentions the shortage: Have you obtained other medications or drugs to deal with these effects? In what way do you feel that your use of medications or drugs has changed? Have you had an overdose since your access to methadone was tapered or stopped?

Final reflections: How has the shortage affected other people you know who were in treatment? How do you feel about taking medication to treat opioid use disorders in general? Has the methadone shortage changed your mind about treatment with medications? How? (Probe: If methadone became more consistently available, would you be interested in continuing to use it? What are the main reasons? If other medicines were available to treat opioid use disorder in Mexico, would you be interested in trying them? What are the names of medicines you have heard out there that you would like to try other than methadone?)

What can the Mexican government do to improve drug treatment services?

In November 2022, the Mexican government closed the operations of the country's methadone production plant and as of April 2023 this resulted in a shortage of methadone throughout the country. We will keep you informed if we know anything about it or if we learn of other solutions that could help you.

Supplemental File 3

Supplemental quotes

Participants' backgrounds

> *"I was around 19 years old, give or take... It was with a guy I was seeing... Uh, we were supposed to go out, but we ended up at his house that time... I saw he was taking things out of his drawer: a spoon and some stuff he was heating up. I didn't know what was happening... I had seen or heard on TV about heroin like that. But I had never seen it before... The guy offered me some and told me it felt amazing... especially during sex, under that dose or substance... And that's the first time I used it... After that time, I only used it for sexual activities two or three times, but later on, not just for sex, but daily, and that's when I became addicted to heroin."* – Daniel (Male, 29)

Introduction to methadone

> *"Because of my job I could not show up all in bad shape and the person who helped me inject was the one who provided me with the solution so I could show up decent and perform well at work. This person told me there's a juice, a miracle juice and so they took me to the clinic, they took me very early in the morning and they gave me the juice…and yes, indeed it was a miracle (juice) because it was really wonderful…well it does work wonders and only for $25 pesos at the time they would give me a (little) cup with the medicine with 150mls or milligrams […] And so, that's how I learned about methadone"* – Andrea (Female, 39)

> *"Yes, it was a while ago. I was using heroin for about two years, and I had problems at home with my family until one day, my mom wanted to take my child away from me because of how I was behaving. I didn't want that, so she helped me find a way through some acquaintances who knew about a treatment back then, which was methadone. She took me to a center—I don't remember exactly, it was ECJ here in Tijuana—and that's when I first started the treatment."* – Laura (Female, 32)

Motivations to use methadone

> *"No, by then he had been detained and sent to jail. I have not had any contact with him since then. Like I was telling you earlier, just recently last year in June I went back to the methadone clinic since there was no reason or anything for me in using heroin."* - Elena (Female, 37)

Experiences of methadone pre-closure

> *"No, the methadone was a good substitute for heroin... I didn't need anything else..."* - Rodrigo (Male, 25)_

> *"They gave me 65 milligrams, but it wasn't enough because I used drugs again, I didn't feel anything, no change, nothing. The next day, they increased the dose to 75 milligrams, and with 75, yes, I felt some sort of strange effect. When I used drugs again, the following day they raised it to 90 milligrams. With those 90, it worked really well."* – Antonio (Male, 49)

"With methadone, you look better—I was showering, feeling better, and the police stopped bothering me. And you know, I decided to get back on methadone, and now, well, they stopped giving it to me." – Manuel (Male, 57)

"I started using drugs again and I was like that for a while and then I started using methadone again, but there came a time when I was using both methadone and heroin at the same time." - Karina (Female, 36)_

"I felt energized, you know? I had a zest for life, wanting to do things, socialize more. Begin to live my life again... I felt more inspired." – Rodrigo (Male, 25)

Closure

"They haven't told us anything…No, no, nothing, who knows why they wouldn't tell us anything, they didn't even put up signs at the clinic or anything…like in other clinics where they sometimes notify you, well, such day, you can come back or whatever…" – Eduardo (Male, 57)

"Honestly, no, they just simply said there's no more program, no more methadone..." – Teresa (Female, 48)

"A friend of mine who goes to the methadone clinic told me that they didn't have any... I didn't believe him... and he was going to come I didn't believe him, I thought he was playing, but then I saw that it's true, that there's none..." – Roberto (Male, 41)

"I signed up, I used it for about two years, and then suddenly they closed the clinic, so I couldn't get methadone anymore because I didn't know where to go. The police were blocking the way to the clinic, saying it was closed. We asked if they were going to sell it again, and they said no, it was going to stay closed. So I didn't want to use heroin anymore; instead, I smoke crystal and take pills now and then." – Manuel (Male, 57)

Post closure

"No, they haven't had a new answer, but what I was seeing is that what happens is that people are drugging with fentanyl, they are replacing it like that." – Karina (Female, 36)_

"Since there's no methadone, I'm on crystal and all that, and that's what I'm working on now. It helps me not to think about the methadone situation because otherwise, I'd be back on methadone". Manuel (Male, 57)

"I used to leave work early because I needed to inject myself, but it's not heroin anymore; it's fentanyl, and it's affecting all of us. If you notice, we all look hunched over; that's what fentanyl does to you. I still look for heroin where I know it's available, you know what I mean? But it's hard to get it." – Román (Male, 55)

Financial impact

“One pill, if it's three a day. Yes, it's affecting my wallet... Yes, and per week, it's much more. A lot more. So, for example, it used to be MXN $40 a day. Mm-hmm. How much is four times seven? Do the math... Sometimes, if I paid for ten days of methadone, it helped. Ten days, that's MXN $400. And yet, in a day, I buy three pills, seven times three, 21 is $210, which means it's affecting everything... It's affecting everything because if I'm going to buy a pill box, I might as well go out, spend another MXN $70 and get another, another MXN $70. And yet, I go to the methadone clinic, I make my way there, pay. Hello, how are you? Greet whoever is there, the security, the secretary... And there's your MXN $40, go get your dose... Stand in line for your dose” – Teresa (Female, 48)

Relationships

“Although I just lost my partner, due to heroin. I had no methadone, and all I wanted was to get rid of withdrawal symptoms, so she said, "If you want to leave, then go," and so I left. It's sad, but I'm telling you, if there was methadone again, I think I could get my life back on track.” – Román (Male, 55)

“Well, there's something that, well... I hope they bring it back so we can continue living more or less normally, because without methadone... You're hardly ever with your family. You're not at home. You use heroin, and I tell you, I go out, get heroin, and then I stay on the streets... I'm out there... Why? Because I want to get the next dose for when I feel like using again…Unlike methadone, it's not like that. I don't get that feeling.” – Teresa (Female, 48)

Material loss and return to criminalization

“Right now, I'm really struggling because there's no methadone. I've already lost my partner due to this situation. And I'm on the verge of losing my job; they told me they'll do a drug test, and I don't know what I'll do. But I hope the clinic opens again before things get worse. I wish someone would do something for the clinics, really, because I'm not the only one going through all this. Many people have lost everything we achieved – the car, the partner – it's sad to go back to heroin, to go back to the streets, to end up in the canal again. We'll all be in the canal again. You can see it now; we, the methadone users, may look changed and clean, but you can see the others are still dirty with their dogs, pushing their carts again. I don't want to go back to that, really. Now that I have a job, I can't go up and down the damn canal. When you're in withdrawal, you don't care if the needle is clean or not; you just need a fix in your desperation for heroin.” – Román (Male, 55)

“Well, I have a little group of friends who used to attend methadone. In fact, I feel sorry for them sometimes because while they were on methadone, they had their money almost clean, and heroin has completely ruined them, right? Ruined them... because they look dirty, they're trying to get their dose, they're trying to get the money... And in contrast, with methadone, it's not like that. Methadone is about work, the dose, work... And it's going really well, right? So, I know two of them, both of them lost their houses: one sold it, and the other one lost it due to, well, heroin, not in a good way, heroin, right? Mm-hmm. In contrast, with methadone, it's the opposite... I mean, heroin is like a dark color, and methadone is, let's say, a light lilac, right? Seeing things as they are... Mm-hmm... In contrast to heroin, no, no, but why? Everything is like this, down there,

because right now, there's no methadone, there's no methadone." – Teresa (Female, 48)

Change in opinion about methadone among participants following the methadone shutdown

"No, it hasn't changed my opinion. For me, personally, the times I've been in treatment, it has positively changed my life and brought about good things" – Antonio (Male, 49)

Policy recommendations for participants

"I think they should consider us, the people who want to have a normal life and stay away from drugs. Methadone helped us, and they should realize that we need it. It's important, and they should take into account the changes it makes in our lives. It's important for them to bring it back and explain to us why it's not available, because they don't tell us anything" – Laura (Female, 32)

"The government needs to step up its efforts. There are many people who need it. Instead of promoting drugs and cartels, they should promote methadone because there's a great need for it. Many people can find relief and change their lives with this treatment." – Antonio (Male, 49)

"Well, I would say that they should obtain methadone again and distribute it, because people will always resort to new drugs, and new drugs are constantly emerging. It would be a way to reduce delinquency, as it was the same thing that led me to use heroin again. So, in that way, it would help reduce drug consumption. Yes, it is necessary to bring back that treatment as a support to stay away from drugs. – Karina (Female, 36)

"Well, you see, sometimes I think the government itself caused the problem by taking away methadone, and now they are the ones creating a big mess. Honestly, I don't think the government is doing anything for us, for the addicts, or for the methadone clinics […]. I don't want to get involved in all of that, and I don't want to talk about things I can't change. I just wish the government could help us, maybe financially or by providing a solution. Can't they make methadone themselves? They have laboratories, right? If they can make crystal meth, can't they make methadone to help us, man? Do you realize where we all ended up again? In the lost city, haven't you heard of it? Only addicts know the suffering we've been through in the lost city after leaving methadone." – Román (Male, 55)

Supplemental File 4

Consolidated criteria for reporting qualitative research (COREQ)

Checklist Item	Guide questions/description	Reported on Page #
	**Domain 1: Research team and reflexivity**	
Personal Characteristics		
1. Interviewer/facilitator	Which author/s conducted the interview or focus group?	Trained field staff conducted interviews, reported on Page 6
2. Credentials	What were the researcher's credentials? E.g. PhD, MD	Title Page
3. Occupation	What was their occupation at the time of the study?	Title Page
4. Gender	Was the researcher male or female?	Not Reported
5. Experience and training	What experience or training did the researcher have?	Not Reported
Relationship with participants		
6. Relationship established	Was a relationship established prior to study commencement?	Page 5
7. Participant knowledge of the interviewer	What did the participants know about the researcher? e.g. personal goals, reasons for doing the research	Page 6
8. Interviewer characteristics	What characteristics were reported about the interviewer/facilitator? e.g. Bias, assumptions, reasons and interests in the research topic	Not Reported, but various biases are reported in limitations on Page 15
	**Domain 2: study design**	
Theoretical framework		
9. Methodological orientation and Theory	What methodological orientation was stated to underpin the study? e.g. grounded theory, discourse analysis, ethnography, phenomenology, content analysis	Page 8
Participant selection		
10. Sampling	How were participants selected? e.g. purposive, convenience, consecutive, snowball	Page 6
11. Method of approach	How were participants approached? e.g. face-to-face, telephone, mail, email	Page 6
12. Sample size	How many participants were in the study?	Page 6

13. Non-participation	How many people refused to participate or dropped out? Reasons?	Not Reported
Setting		
14. Setting of data collection	Where was the data collected? e.g. home, clinic, workplace	Page 5 and 6
15. Presence of non-participants	Was anyone else present besides the participants and researchers?	Page 6
16. Description of sample	What are the important characteristics of the sample? e.g. demographic data, date	Page 10
Data collection		
17. Interview guide	Were questions, prompts, guides provided by the authors? Was it pilot tested?	Page 8-9, Supplemental Table 1
18. Repeat interviews	Were repeat interviews carried out? If yes, how many?	No repeat interviews were conducted
19. Audio/visual recording	Did the research use audio or visual recording to collect the data?	Page 6
20. Field notes	Were field notes made during and/or after the interview or focus group?	Field notes were not taken
21. Duration	What was the duration of the interviews or focus group?	Page 6
22. Data saturation	Was data saturation discussed?	Page 6
23. Transcripts returned	Were transcripts returned to participants for comment and/or correction?	Transcripts were not returned
	**Domain 3: analysis and findings**	
Data analysis		
24. Number of data coders	How many data coders coded the data?	Page 7
25. Description of the coding tree	Did authors provide a description of the coding tree?	Table 1
26. Derivation of themes	Were themes identified in advance or derived from the data?	Pages 6 and 7
27. Software	What software, if applicable, was used to manage the data?	Page 7
28. Participant checking	Did participants provide feedback on the findings?	No, participants did not provide feedback
Reporting		
29. Quotations presented		

	Were participant quotations presented to illustrate the themes/findings? Was each quotation identified? e.g. participant number	Page 11 through 14, Appendix
30. Data and findings consistent	Was there consistency between the data presented and the findings?	Data presented and findings discussed are in alignment
31. Clarity of major themes	Were major themes clearly presented in the findings?	Pages 11 through 14
32. Clarity of minor themes	Is there a description of diverse cases or discussion of minor themes?	Pages 11 through 14
